# Endoscopic removal of a gastric pharmacobezoar induced by clomipramine, lorazepam, and domperidone overdose: a case report

**DOI:** 10.1186/s13256-019-1984-0

**Published:** 2019-02-27

**Authors:** Stephan von Düring, Corinne Challet, Laurent Christin

**Affiliations:** 1 Department of Internal Medicine, Emergency Medicine and Critical Care Medicine, Groupement Hospitalier de l’Ouest Lémanique (GHOL), Nyon Hospital, Chemin Monastier 10, 1260 Nyon, Switzerland; 2 Department of Pharmacy, Pharmacie Interhospitalière de la Côte, Chemin du Crêt 2, 1110 Morges, Switzerland

**Keywords:** Pharmacobezoar, Clomipramine, Acute tricyclic antidepressant poisoning, Drug overdose, Sustained-release formulation

## Abstract

**Introduction:**

Gastric pharmacobezoars are a rare entity that can induce mechanical gastric outlet obstructions and sometimes prolong toxic pharmacological effects. Certain medications, such as sustained-release forms, contain cellulose derivatives that may contribute to the adhesion between pills and lead to the creation of an aggregate resulting in a pharmacobezoar. Case reports are rare, and official guidelines are needed to help medical teams choose proper treatment options.

**Case presentation:**

Our patient was a 40-year-old Caucasian woman with borderline personality disorder and active suicidal thoughts who was found unconscious after a massive drug consumption of slow-release clomipramine, lorazepam, and domperidone. On her arrival in the emergency room, endotracheal intubation was preformed to protect her airway, and a chest x-ray revealed multiple coffee grain-sized opaque masses in the stomach. She was treated with activated charcoal followed by two endoscopic gastric decontaminations 12 h apart in order to extract a massive gastric pharmacobezoar by manual removal of the tablets.

**Conclusion:**

This case demonstrates that in the case of a massive drug consumption, a pharmacobezoar should be suspected, particularly when cellulose-coated pills are ingested. Severe poisoning due to delayed drug release from the gastric aggregate is a potential complication. Detection by x-ray is crucial, and treatment is centered on removal of the aggregate. The technique of decontamination varies among experts, and no formal recommendations exist to date. It seems reasonable that endoscopic evaluation should be performed in order to determine the appropriate technique of decontamination. Care should be patient-oriented and take into account the clinical presentation and any organ failure, and it should not be determined solely by the suspected medication ingested. Thus, serum levels are not sufficient to guide management of tricyclic antidepressant intoxication.

## Background

A gastric bezoar results from an aggregation of foreign bodies in the stomach. The major types are phytobezoars (vegetable matter), trichobezoars (hair), and pharmacobezoars (ingested drugs) [1]. Pharmacobezoars are a rare entity and result from an aggregation of pills ingested at the same time. Certain substances are more likely to bond than others [2]. All types of gastric bezoars can induce mechanical gastric outlet obstruction, but pharmacobezoars also present the risk of a prolonged drug release with multiple peak plasma concentrations and an increased risk of toxicity [3].

It is difficult to know when to suspect a pharmacobezoar, but they should always be considered in cases of massive drug consumption. Clinical presentations are not always helpful. Abdominal x-ray may be helpful, but only when the tablets are radio-opaque.

In this case report, we discuss the proper management of multidrug gastric pharmacobezoars, because they are a rare entity and no formal guidelines exist on how to deal with them. Our patient’s case demonstrates that abdominal x-rays can be helpful to confirm initial suspicions, but an early gastric endoscopy is necessary for the diagnosis and management and furthermore must be repeated until full extraction is achieved.

## Case presentation

**Fig. 1:**
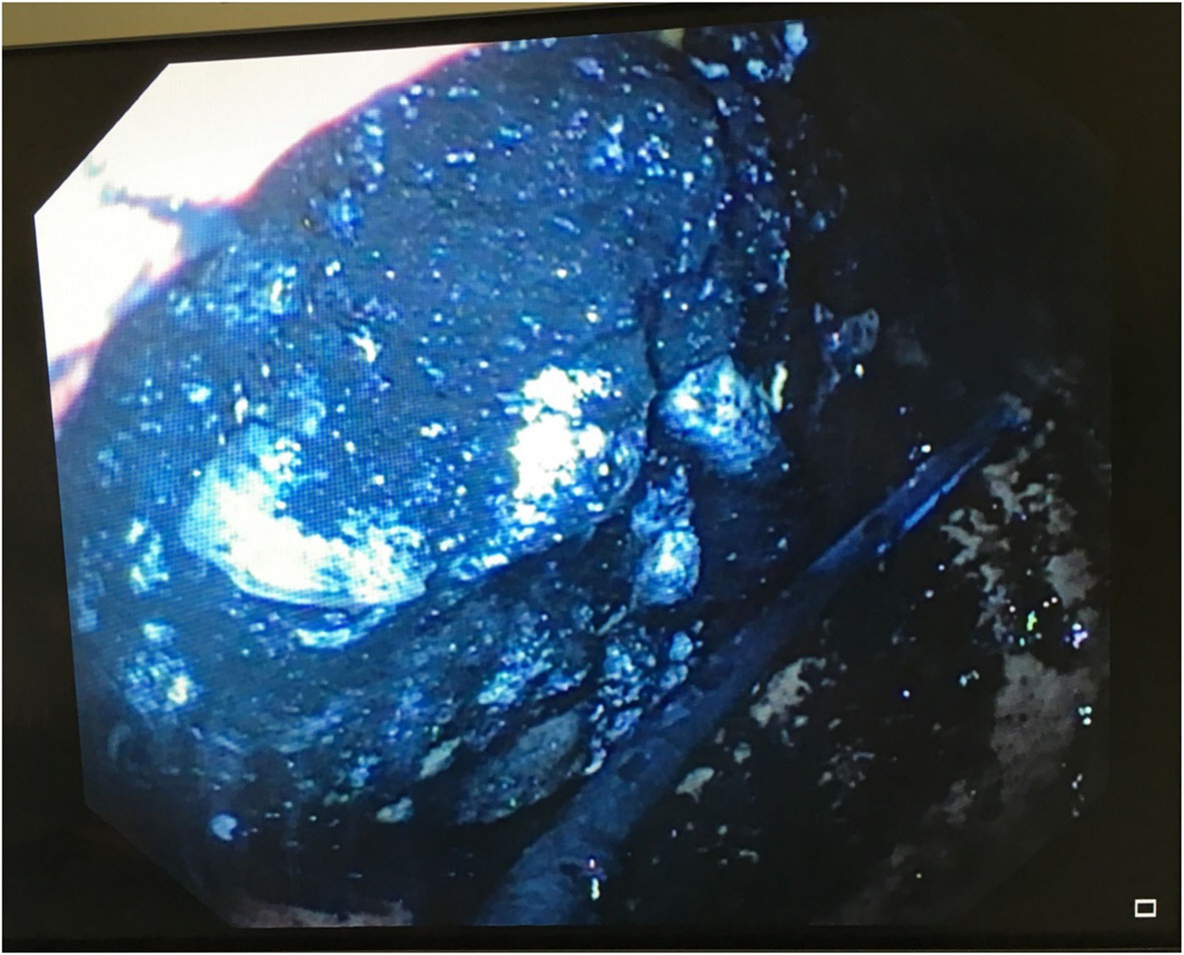
Gastric endoscopy shows pharmacobezoar extending from the fundus to the great curvature of the stomach after activated charcoal decontamination with intact and broken tablets

**Fig. 2:**
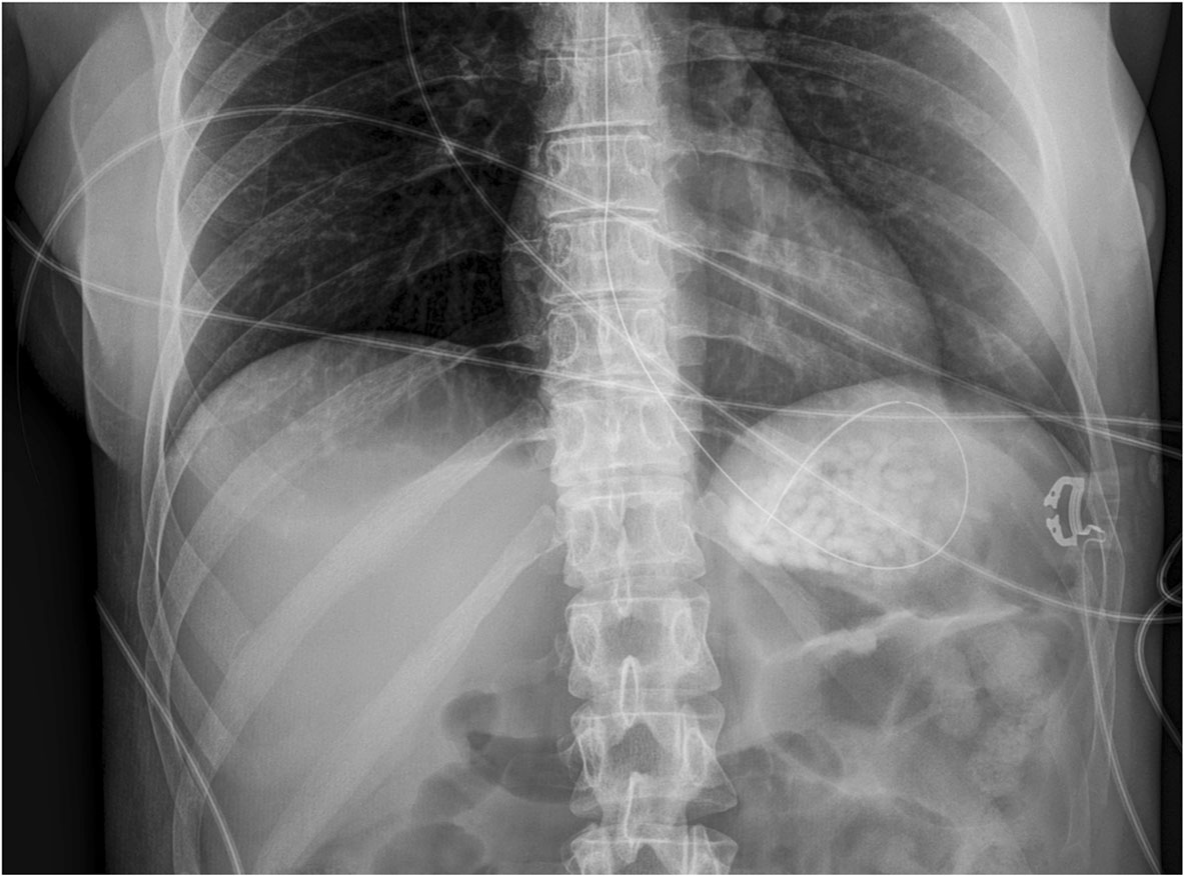
Abdominal x-ray confirming presence of gastric pharmacobezoar

A 40-year-old Caucasian woman with training in human resources but unemployed since 2014 due to borderline personality disorder and active suicidal thoughts, was found unconscious at home by her husband. She was a nonsmoker and a social drinker. Her medical treatment consisted of clomipramine 150 mg once daily and lorazepam 2.5 mg twice daily. She was rapidly transported to the emergency room (ER) by ambulance with an oxygen mask. On arrival, her vital signs were as follows: blood pressure of 119/62 mmHg, heart rate of 62 beats/min, and temperature of 35.0 °C. She was unalert with a Glasgow Coma Scale score of 5/15 (E1 V1 M3) and presented no protective airway reflexes. The result of her cardiopulmonary examination was normal, and we found no abdominal distention or guarding and no masses on palpation. Neurological examination revealed an unconscious patient with a slight reactive bilateral miosis and no focal neurological deficits on cranial nerve or peripheral neurological examination. Laboratory findings were within normal range, including a complete blood count (hemoglobin of 133 g/L, white cell count of 6.2 × 10^9^/L, platelet count of 153 × 10^9^/L), coagulation test, full electrolytes, kidney and liver function tests. Arterial blood gas showed a nonhypoxemic respiratory acidosis (pH 7.34, partial pressure of oxygen 56.9 kPa, partial pressure of carbon dioxide 6.2 kPa, bicarbonate 24.8 mmol/L). We proceeded to perform an endotracheal intubation (propofol 50 mg, fentanyl 50 μg, suxamethonium chloride 70 mg, rocuronium 50 mg, propofol 100 mg/h, and a slow drip of 250 ml of Ringer’s lactate solution) followed by a chest x-ray that revealed multiple coffee grain-sized opaque masses in the stomach. Empty blister packs found around her by paramedics suggested an ingestion of up to 8,625 g of slow-release clomipramine (Anafranil SR® 75 mg; Novartis Pharma Schweiz AG, Switzerland), 125 mg of lorazepam (Temesta® 2.5 mg; Pfizer PFE Switzerland GmbH), and 160 mg of domperidone (Motilium® 10 mg; Janssen-Cilag AG). In accordance with the national poisons information center (Tox Info Suisse, Zürich, Switzerland), we started a multidose activated charcoal (AC) regimen (60 g loading dose in the ER, completed by 30 g every 6 h for 24 h), followed by a gastric endoscopy that found an important pharmacobezoar extending from the fundus to the great curvature of the stomach (Fig. 1). Three liters of normal saline were used in the stomach to fragment the aggregate, and a manual extraction of the tablets was performed with a wire basket with partial success. The patient was admitted to the intensive care unit (ICU) for mechanical ventilation and further observation, and she never showed any signs of cardiovascular disturbance. Her treatment in the ICU consisted of fentanyl 0–50 μg/h, enoxaparin 40 mg subcutaneously once daily, AC 30 g every 6 h, and glucosaline intravenous drip 1 L/day. Eight hours after ICU admission, another abdominal x-ray (Fig. 2) confirmed a persistent gastric tablet aggregate, and a second attempt at gastroscopic extraction was performed. The patient gradually awakened and was weaned off mechanical ventilation after 30 h. She was transferred to our psychiatric unit on day 3 for further care. During her stay, she had normal electrolytes, hemoglobin, and white blood cell count. She never presented any hemodynamic instability, had no QT interval prolongation on electrocardiogram or any arrhythmias, and we had no clinical argument for seizures. No therapeutic drug monitoring of clomipramine was performed at baseline or during the patient’s hospital stay because she improved and recognized having taken all the tablets from the empty blister packs found at her home. She was hospitalized in our local psychiatric hospital and discharged after 10 days. Regular follow-up with her psychiatrist was uneventful for the next 2 years.

## Discussion

This patient was brought to our ER in a comatose state after a massive drug intake. She was treated for a gastric pharmacobezoar, and her evolution was favorable. Despite bezoar removal, the treatment of this patient was based essentially on supportive care. Unlike some papers which suggest that tricyclic antidepressant (TCA) serum levels are needed to guide therapy, we believe that this is not patient-oriented care because TCA serum levels correlate poorly with outcome. In general, treatment should be based on the known properties of the ingested substance(s), the clinical presentations, and the organ failures. Serum levels should be considered solely when they impact the treatment (for example, paracetamol intoxication). To our knowledge, this concept of care is rarely promoted but is essential and in line with the current “less is more” movement of 21st-century medicine.

Several factors may have contributed to the pharmacobezoar formation in our patient, including massive tablet ingestion (estimated at 181 tablets) and clomipramine’s characteristics (Table 1). In addition to their inhibition of norepinephrine and serotonin reuptake, TCAs possess antihistaminic and anticholinergic effects [4, 5]. This latter can impair gastric motility, compromising gastric emptying and favoring bezoar formation in cases where concomitant predisposing factors are present. Moreover, the clomipramine ingested by our patient was a sustained-release formulation (Anafranil SR®) containing hypromellose, a cellulose derivative. We suggest that this compound may have formed a gel-like layer in the stomach and could be responsible for the adhesion with other pills when taken together, and that it thereby provoked an aggregation resulting in a pharmacobezoar, in a similar manner to cellulose acetate [6].

**Table 1:** Characteristics and risks related to overdose of the drugs implicated in this case

Drug name	Maximal oral daily dosing (adult) 1	Half-life (adult)	Intoxication signs (non-exhaustive list) 2	Risk factors for bezoar’s formation in the reported case
Clomipramine	250 mg/d	• Clomipramine: 19 - 37 h • Desmethylclomipramine (active metabolite): 54 – 77 h	• Tachycardia, hypotension, orthostasis • PR, QRS, QTc intervals prolongation • Anticholinergic effects (*e.g.* blurred vision, delirium, agitation, hallucinations, mydriasis, sinus tachycardia, urinary retention) • Seizures, sedation	• Anticholinergic effect • Sustained-release formulation with hypromellose component • Massive tablets ingestion (*i.e.* 115 tablets, 8,625 g)
Domperidone	30 mg/d	~ 7 h	• QT interval prolongation, cardiac arrhythmia • Restlessness, drowsiness, insomnia, headache, confusion, dizziness and acute dystonic reactions, akathisia, parkinsonian-like symptoms • Diarrhea, dry mouth	• Massive tablet ingestion (*i.e.* 16 tablets, 160 mg)
Lorazepam	10 mg/d	~ 12 h	• Sedation, dizziness, amnesia, ataxia, slurred speech, lethargy • Hypotension • Hypothermia, respiratory failure • Coma	• Massive tablet ingestion (*i.e.* 50 tablets, 125 mg)

References:

[1] Lexicomp mobile software, Lexi-Drugs, Hudson, Ohio: Wolters Kluwer Clinical Drug Information, Inc., July 14, 2016

[2] Lexicomp mobile software, Lexi-Tox, Hudson, Ohio: Wolters Kluwer Clinical Drug Information, Inc., July 14, 2016

According to the GEMNet TCA overdose guidelines, AC may be considered within the first hour of TCA ingestion to reduce drug absorption and bioavailability, but there is no substantial evidence that it is of benefit [5]. Multiple doses should not be continued thereafter, owing to the risk of pulmonary aspiration due to the delayed gastric emptying. Some authors suspect that AC itself could also exacerbate pharmacobezoar formation by participating in the adhesion process [1]. No specific antidote to TCA exists.

Endoscopic gastric decontamination and pill removal is the method of choice to manage a bezoar. However, the repetition of required gastroscopies can result in gastric hemorrhages and be deleterious. [7, 8]. In order to fragment the pill aggregate for a proper retrieval, a gastroscopic water-jet fragmentation can be performed for aspiration or basket retrieval. A risk encountered with this approach could be a larger release of active drug from the conglomerate and an increased absorption associated with toxicity. Thus, its pros and cons, as well as the patient’s clinical status, must be evaluated before proceeding. Some authors have used the acid proprieties of soft drinks to dissolve a bezoar, but to our reasoning this can only be used in non-imminently lethal cases with a type of bezoar that can be dissolved by acid [9]. Surgical removal of the bezoar may be necessary in some cases but was not considered for our patient, owing to the good evolution she demonstrated.

Seizures can occur with TCA overdose, usually beginning early after ingestion, and are usually brief, self-limited, generalized tonic-clonic seizures [10]. The underlying mechanism could be related to the antagonist effects of TCAs on the γ-aminobutyric acid type A receptor. Our patient presented no sign of seizures during hospitalization, probably in part due to the 125 mg of lorazepam she had ingested simultaneously. TCA can induce marked cardiovascular deterioration [11, 12]. Rhabdomyolysis has also been described in rare cases, but the mechanism remains unclear [13]. These complications were not observed in our patient.

## Conclusion

Our patient was admitted for a drug overdose of slow-release clomipramine, lorazepam, and domperidone resulting in a gastric pharmacobezoar, and she was treated by AC and endoscopic gastric decontamination. The massive tablet ingestion, as well as the sustained-release form of clomipramine, contributed to bezoar formation [1, 7]. Moreover, the anticholinergic properties of clomipramine may have decreased gastrointestinal motility, counteracting the effect of domperidone.

In any case of drug overdose, a pharmacobezoar should be suspected, especially when cellulose-coated pills are ingested. Because severe poisoning due to delayed drug release from the gastric aggregate is a potential complication, detection by x-ray is a reasonable first examination [3, 8]. Endoscopic evaluation is mandatory, and management by endoscopic or surgical removal should be considered rapidly.

Serum levels are not useful to guide management of TCA intoxication because they correlate poorly with clinical effects. They do not help to predict toxicity, even if high serum levels are usually associated with severe toxicity. A qualitative test may be performed to confirm an exposure to a TCA if suspected. False-positive results of urine TCA screenings are common.

In light of the wide variety of drugs often implicated in drug overdoses, our patient’s case demonstrates why patient-oriented care should not be based solely on the pharmacological properties of the individual substances. Several factors play a role in patient care: the combination of known chemicals ingested, the clinical presentation corresponding to the pharmacological effect, and the organ failure induced. It seems reasonable to assume that our patient’s favorable outcome was due to the rapid partial removal of the pharmacobezoar and to the AC regimen, even if this latter treatment is controversial in the case of bezoars.

## Abbreviations

AC: Activated charcoal
ER: Emergency room
ICU: Intensive care unit
TCA: Tricyclic antidepressants
